# Assessment of Medication Adherence and Its Demographic and Clinical Determinants After Coronary Artery Bypass Grafting

**DOI:** 10.7759/cureus.89909

**Published:** 2025-08-12

**Authors:** Kyriakos Alexandrou, Nicos Middleton, Maria Kyranou, Pavlos Sarafis

**Affiliations:** 1 Department of Nursing, Cyprus University of Technology, Limassol, CYP; 2 Department of Nursing, University of Thessaly, Lamia, GRC

**Keywords:** cardiac surgery, cardiovascular patients, coronary artery bypass grafting, medication adherence, postoperative care

## Abstract

Introduction

Medication adherence is a critical component of postoperative care in cardiac surgery patients. Nonadherence may compromise recovery and long-term outcomes. This study aimed to measure medication adherence at three and six months postoperatively and examine its association with demographic and clinical factors among patients undergoing coronary artery bypass grafting (CABG) in Cyprus.

Materials and methods

This prospective multicenter study was conducted in two private hospitals and one public hospital in Cyprus between September 2022 and April 2023. A total of 97 adult patients who underwent elective CABG and completed follow-up assessments were included in the final analysis. Medication adherence was evaluated at three and six months postoperatively via structured telephone interviews using the Adherence to Refills and Medications Scale (ARMS). Demographic and clinical data were collected from medical records and preoperative interviews. Non-parametric tests (Wilcoxon, Mann-Whitney U, Kruskal-Wallis) were applied to examine adherence over time and its associations with demographic and clinical factors.

Results

The ARMS score significantly decreased from 13.24 (±1.34) at three months to 12.85 (±1.16) at six months (median: 13.00 to 12.00; p=0.012), indicating improved medication adherence over time. The proportion of patients with high adherence increased from 37.1% at three months to 50.5% at six months. Analysis of demographic and clinical factors showed that male gender (p=0.043) and the presence of diabetes mellitus (p=0.025) were significantly associated with higher adherence levels at six months.

Discussion

This study demonstrated a significant improvement in medication adherence over the first six months following CABG, particularly among male patients and those with diabetes mellitus. The improvement occurred in the absence of structured interventions, suggesting that surgery may serve as a motivational trigger for behavioral change. However, the persistence of moderate adherence highlights the need for long-term, patient-centered strategies to support sustained medication adherence in this population.

## Introduction

Coronary artery bypass grafting (CABG) is a widely established cardiac surgery technique and one of the most commonly performed interventions worldwide [1,2]. It provides significant long-term benefits in survival and improving symptoms in patients with multivessel disease and has a high success rate [2,3].

Successful outcomes after CABG depend not only on the surgical procedure itself but also on the long-term adherence of patients to their pharmacological treatment [1,4]. The therapeutic regimen includes, among others, antiplatelets, beta-blockers, statins, and angiotensin-converting enzyme (ACE) inhibitors, aiming to prevent complications and improve prognosis. However, medication adherence often remains insufficient [2], and its clinical importance is frequently underestimated. Medication adherence refers to the extent to which patients take their prescribed medications consistently and correctly, as advised by their healthcare providers [5].

Sub-optimal adherence is associated with an increased risk of complications, readmissions, and mortality [6,7]. Globally, up to 50% of patients with chronic diseases do not follow their pharmacological treatment as prescribed, which burdens healthcare systems [8]. In this study, medication adherence was evaluated both as a continuous score using the Adherence to Refills and Medications Scale (ARMS) scale and as a categorical variable, classified into high, moderate, and low levels of adherence, as described in the Methods section. Approximately one in five patients who undergo CABG demonstrate sub-optimal medication adherence within six months postoperatively [5]. In patients who have undergone CABG, sub-optimal adherence has been associated with an increased risk of myocardial infarction and death, while good adherence is associated with higher survival rates and improved quality of life [5]. The causes of sub-optimal adherence are multifactorial and include demographic characteristics, clinical parameters, perceptions about treatment, psychological status, and the level of knowledge about the disease and pharmacotherapy [9,10].

Despite clear recommendations for long-term pharmacological treatment after CABG, non-adherence remains a "silent barrier" to the success of secondary prevention [7]. Investigating the factors associated with adherence is essential for designing targeted interventions that will improve clinical outcomes and reduce adverse complications [8].

In the Cypriot population as well as in the international literature, data on adherence after CABG and related factors remain limited, possibly due to the predominant focus on surgical outcomes and short-term complications rather than long-term adherence patterns. To our knowledge, no previous study has examined medication adherence after CABG in Cyprus. This study aims to assess the level of medication adherence at both three and six months postoperatively and to investigate the demographic and clinical factors associated with adherence among patients after CABG in Cyprus.

## Materials and methods

Study design

This is the first prospective multicenter study conducted in Cyprus with the aim of investigating medication adherence in patients following CABG. Data collection took place in three hospitals where CABG procedures are performed: two private hospitals and one public hospital. These hospitals are located in the two largest cities of the country but are visited by patients from all districts. The multicenter approach was chosen to enhance the representativeness and generalizability of the findings.

Study population

A total of 97 patients were included in the final analysis. Initially, 111 adult patients scheduled for elective CABG were approached to participate in the study. Of these, 102 patients provided informed consent and completed baseline data collection prior to surgery. Inclusion criteria included age ≥18 years and eligibility for scheduled surgical intervention. Patients who were scheduled for emergency CABG or were in critical clinical condition, such as being intubated, sedated, unconscious, or otherwise unable to provide informed consent, were excluded. Additionally, individuals who could not communicate in either Greek or English were also to be excluded; however, no such cases occurred during the recruitment process. As part of the baseline data collection, the type of surgery (isolated CABG or CABG combined with valve procedure) was also recorded and considered for subsequent subgroup analyses.

A power analysis indicated that a minimum of 56 participants would be required to detect a within-subject effect size of 0.8, with 90% power and a 5% significance level. To ensure statistical robustness and account for potential attrition, the sample was intentionally enlarged. After accounting for loss to follow-up or incomplete data, 97 patients were included in the final analysis, representing approximately 60.4% of all elective CABG procedures performed at the participating hospitals during the study period.

A convenience sampling method was used due to time and resource constraints. Of the 111 patients invited to participate, 102 (91.9%) provided written informed consent (80 men, 22 women). Five patients did not complete follow-up (four due to loss of contact and one due to death). The final sample for the analysis of medication adherence included 97 participants.

Data collection

Data collection was conducted by trained nurses who were members of the research team and had received specific instruction on standardized procedures to ensure consistency across all sites. Baseline demographic and clinical data were obtained from patients’ medical records and through direct preoperative interviews conducted in person. Medication adherence was assessed at two postoperative time points: three months and six months after hospital discharge. These assessments were conducted via structured telephone interviews using the ARMS. The same trained nurses carried out all telephone assessments, following uniform guidance developed by the research team to minimize interviewer bias and ensure methodological consistency. To minimize interviewer bias, the nurses conducting the telephone interviews were blinded to participants’ previous responses and baseline characteristics.

Study instruments

Sociodemographic data were collected by trained nurses through direct preoperative interviews using a standardized data collection form. The collected variables included age, gender, educational level, and marital status. Clinical variables were extracted from patients’ medical records and included medical history (such as hypertension, dyslipidemia, diabetes mellitus, myocardial infarction), presence of arrhythmias, left ventricular ejection fraction (LVEF), number of grafts, and type of surgery (isolated CABG or combined procedure). Length of ICU stay and total hospital stay were also recorded from medical charts as indicators of postoperative recovery.

Medication adherence was assessed using the ARMS. The ARMS was developed at Emory University as a simple and user-friendly tool for assessing adherence, suitable for all patients, including people with lower educational level or limited reading skills, and is the only relevant tool addressing cardiac surgery patients [11]. Although the ARMS had previously been used in Greek-speaking populations, it had not been applied in Cyprus before; therefore, it was pilot-tested on a small sample to ensure clarity and cultural appropriateness, with no modifications deemed necessary.

The ARMS consists of 12 items covering both medication intake and refill behaviors, as well as exploring potential barriers affecting these processes. Responses are provided on a 4-point Likert scale (“Never,” “Sometimes,” “Most of the time,” “Always”). The total score is the sum of the responses, with higher scores indicating lower adherence. According to ARMS scoring, high adherence is defined as a total score of 12, moderate adherence as a score between 13 and 34, and low adherence as a score greater than 34 [11].

The reliability and validity of the ARMS scale have been reported in its original validation study. Internal consistency (Cronbach’s alpha) was 0.81 overall, 0.79 for individuals with low educational level, and 0.82 for those with moderate educational level. Its concurrent validity was demonstrated through a strong correlation with the Morisky scale [11].

Ethical considerations

Τhe study was conducted in accordance with the ethical standards outlined in the Declaration of Helsinki and was approved by the Cyprus National Bioethics Committee (approval number: 2021.01.153) and the State Health Services Organization. Additional administrative approvals were obtained from the directors of all three participating hospitals.

Patients were informed about the study during the pre-surgical period through face-to-face communication with trained clinical nurses and members of the research team. Detailed verbal and written information was provided, explaining the purpose of the study, the assessments involved, potential risks and benefits, data confidentiality, and the voluntary nature of participation. Patients were given sufficient time to ask questions and consider their participation.

Written informed consent was obtained prior to surgery and before any data collection began. Participants were also informed of their right to withdraw from the study at any point without any impact on the care they received.

Statistical analysis

Data analysis was performed using SPSS Statistics, Version 29.0 (IBM Corp., Armonk, NY). The normality of the distribution of continuous variables was initially tested using the Shapiro-Wilk test. Since the variables did not follow a normal distribution, non-parametric tests were applied. The Wilcoxon signed-rank test was used to compare adherence levels at three and six months. Comparison of adherence scores between demographic and clinical subgroups was conducted using the Mann-Whitney U test (for two groups) and the Kruskal-Wallis test (for more than two groups). The level of statistical significance was set at p<0.05.

## Results

Age and type of surgery by gender

A total of 102 patients who underwent CABG participated in the collection of clinical and demographic data during the preoperative period. During follow-up, four participants were unreachable, and one patient died in the ICU, leaving 97 participants for the final analysis. Medication adherence was assessed exclusively in these 97 participants who completed follow-up, ensuring consistency in the analysis population. The age of participants ranged from 38 to 84 years, with a mean age of 66.8 years (SD: 9.09) and a median of 68 years (IQR: 63-74). The majority were male (n=80, 78.4%).

Most men were aged 60 years or older, with 38.8% in the age range of 60-69 years and 42.5% aged ≥70 years. Similarly, 86.4% of women were aged 60 or above. Isolated CABG was performed in 70% of male and 50% of female patients, while the remainder underwent combined procedures involving valve surgery (Table 1).

**Table 1:** Age and type of surgery by gender AV, aortic valve; CABG, coronary artery bypass grafting; MV, mitral valve

Age and type of surgery	Male, n=80	Female, n=22
	n (%)	n (%)
Age (years)		
≤59	15 (18.7)	3 (13.6)
60-69	31 (38.8)	8 (36.4)
≥70	34 (42.5)	11 (50)
Type of surgery		
CABG	56 (70.0)	11 (50.0)
CABG+AV repair	5 (6.3)	5 (22.7)
CABG+AV replacement	9 (11.3)	2 (9.1)
CABG+MV repair	2 (2.4)	1 (4.5)
CABG+MV replacement	8 (10.0)	3 (13.6)

Clinical characteristics by gender

Common comorbidities in the total sample included hypercholesterolemia (66.7%, n=68), hypertension (59.8%, n=61), diabetes mellitus (45.1%, n=46), and heart failure (24.5%, n=25). The mean LVEF was 49.2% (SD: 8.8), with a median of 50% (IQR: 45-55).

Among men, 60% had hypertension, 65% had dyslipidemia, and 42.5% had diabetes mellitus. In women, the corresponding percentages were 59.1%, 72.7%, and 54.6%, respectively. Additionally, 60% of male patients and 72.7% of female patients had an LVEF of ≤50% (Table 2). Recorded complications included arrhythmias (26.5%, n=27), primarily atrial fibrillation (AF) (20.6%, n=21), and acute renal failure requiring dialysis (2.9%, n=3). The mean ICU stay was 1.78 days (SD: 6.84), with a median of 1 day, while the mean hospital stay was 8.94 days (SD: 11.48), with a median of 6 days.

**Table 2:** Clinical characteristics by gender LVEF, left ventricular ejection fraction

Clinical characteristics	Male, n=80	Female, n=22
	n (%)	n (%)
Age (years)		
≤59	15 (18.7)	3 (13.6)
60-69	31 (38.8)	8 (36.4)
≥70	34 (42.5)	11 (50)
Hypertension		
Yes	48 (60)	13 (59.1)
No	32 (40)	9 (40.9)
Dyslipidemia		
Yes	52 (65)	16 (72.7)
No	28 (35)	6 (27.3)
Diabetes		
Yes	34 (42.5)	12 (54.6)
No	46 (57.5)	10 (45.4)
LVEF		
LVEF ≤40%	12 (15.0)	7 (31.8)
LVEF 41-50%	36 (45.0)	9 (40.9)
LVEF >50%	32 (40.0)	6 (27.3)

Medication adherence after surgery

**Figure 1:**
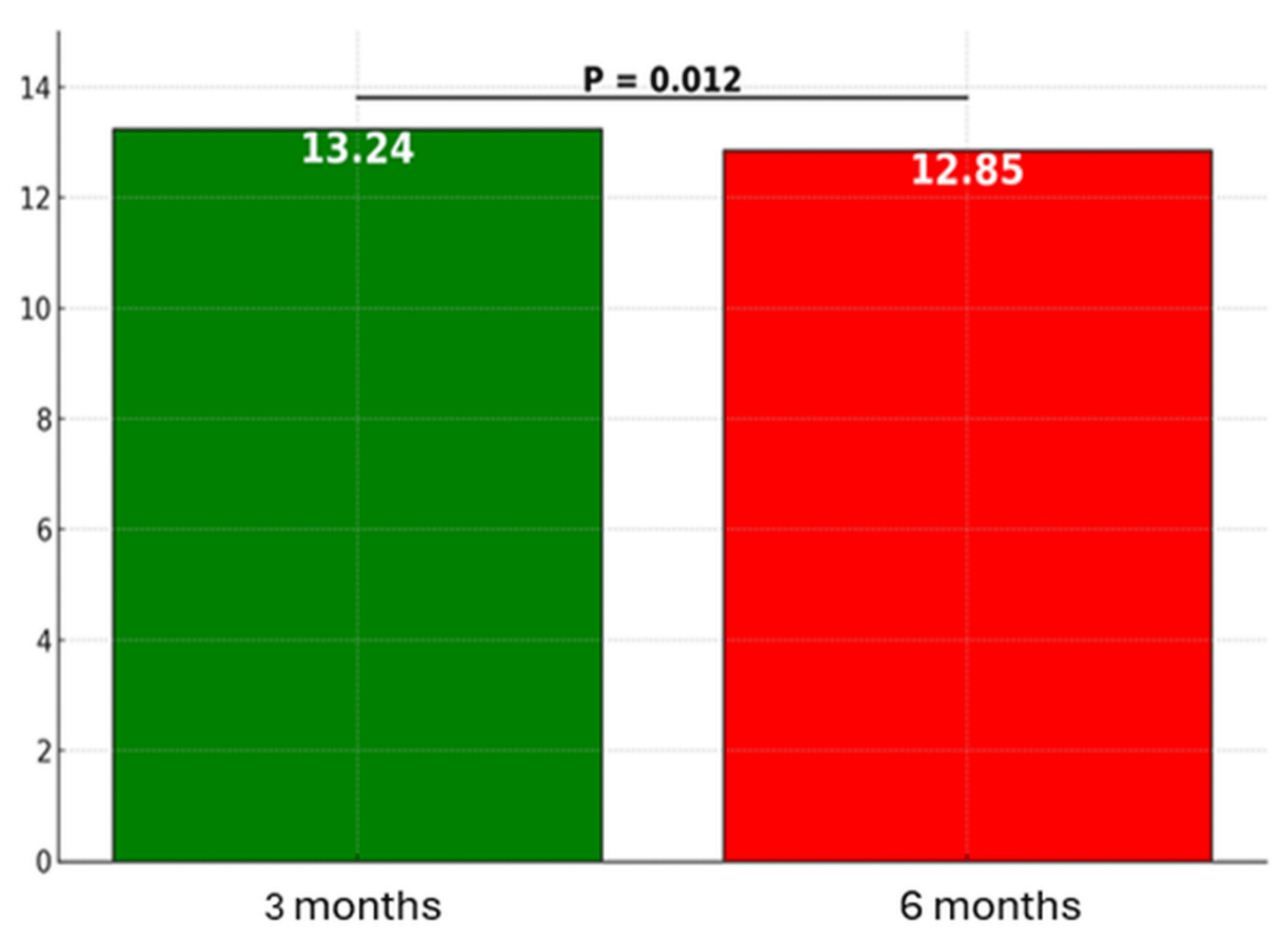
Change in mean ARMS scores at three and six months post-CABG ARMS, Adherence to Refills and Medications Scale; CABG, coronary artery bypass grafting

At three months, the mean ARMS score was 13.24 (SD ±1.34), with a median of 13. Although the theoretical range of the ARMS scale is 12-48, the observed scores in this sample ranged from 12 to 18, reflecting a narrow distribution and overall high adherence. At six months, the mean ARMS score further decreased to 12.85 (SD ±1.16), with a median of 12 and the same observed range (12-18). This reduction was statistically significant based on the Wilcoxon signed-rank test (p = 0.012), indicating an improvement in medication adherence over time (Figure 1). Since lower ARMS scores correspond to higher levels of adherence, this finding demonstrates that patients showed greater consistency and commitment to their prescribed medication regimen at six months postoperatively.

The reduction in both the mean and median scores, combined with the smaller standard deviation at six months, indicates a more homogeneous and consistently improved pattern of adherence among patients at the second time point. The ARMS scores at both assessments suggest that most participants demonstrated relatively high levels of adherence throughout the postoperative follow-up period. Detailed data are presented in Table 3. Although an overall improvement in patient adherence is observed, as reflected by the decrease in the mean ARMS score from 13.24 at three months to 12.85 at six months, it is noteworthy that a significant proportion of participants continued to fall into the moderate adherence category at both time points. Specifically, at three months, 62.89% of patients showed a moderate level of adherence, while at six months, this proportion decreased to 49.5%. A detailed summary of these findings is presented in Table 3.

**Table 3:** Medication adherence scores and levels at three and six months Theoretical range: 12–48, with lower scores indicating better medication adherence. ARMS, Adherence to Refills and Medications Scale; SD, standard deviation

ARMS		
Time	3 months	6 months
Participants	97	97
Mean	13.24	12.85
Median	13	12.00
SD	±1.34	±1.16
Range	6	6
Minimum	12	12
Maximum	18	18
High (12)	36 (37.11)	49 (50.5)
Moderate (13-34)	61 (62.89)	48 (49.5)
Low (>34)	0 (0)	0 (0)

Demographic factors associated with medication adherence

The investigation of demographic factors potentially associated with postoperative medication adherence revealed that most variables did not show a statistically significant association. However, participant gender was significantly associated with adherence at both three months (p=0.0299) and six months (p=0.043). At three months, male participants had a median ARMS score of 13.00 (mean: 13.12 ± 1.20), while female participants also had a median of 13.00 (mean: 13.54 ± 1.60). At six months, the median score was 12.00 for males (mean: 12.76 ± 1.18) and 13.00 for females (mean: 13.13 ± 1.04), indicating better adherence among male participants at both time points. Detailed adherence data across demographic categories, including central tendency measures and statistical comparisons, are presented in Table 4.

**Table 4:** Association of demographic factors with medication adherence at three and six months postoperatively Statistical tests: *Mann-Whitney U test; **Kruskal-Wallis H test.

Variable	Category	3 months, mean (SD)	3 months, median [IQR]	6 months, mean (SD)	6 months, median [IQR]	p-value and test (3 months)	p-value and test (6 months)
Gender*	Male	13.12 (±1.20)	13.00 [12.00–14.00]	12.76 (±1.18)	12.00 [12.00–13.00]	0.0299; test=709.5	0.043; test=610.5
	Female	13.54 (±1.60)	13.00 [12.00–14.25]	13.13 (±1.04)	13.00 [12.00–14.00]		
Employment status*	Employed	13.33 (±1.38)	13 [12.00-14.00]	13.00 (±1.33)	13.00 [12.00-14.00]	0.547; test=1,081.5	0.361; test=1,046
	Unemployed	13.13 (±1.24)	13 [12.00–1400]	12.72 (±0.99)	12.00 [12.00-13.00–]		
Age group**	≤59	13.72 (±1.74)	13 [12.00-15.00]	13.17 (±1.30)	13 [12.00-14.00]	0.319; test=2.284	0.457; test=1.566
	60-69	13.19 (±1.17)	13 [12.00-14.00]	12.78 (±1.23)	12 [12.00-13.00]		
	≥70	13.02 (±1.16)	13 [12.00-14.00]	12.76 (±1.03)	12 [12.00-13.00]		
Educational level**	Primary	13.38 (±1.58)	13 [12.00-14.00]	12.96 (±1.23)	12 [12.00-14.00]	0.493; test=1.416	0.281; test=2.538
	Secondary	13.29 (±1.26)	13 [12.00-14.00]	12.91 (±1.17)	13 [12.00-13.00]		
	Tertiary	12.92 (±1.06)	13 [12.00-13.00]	12.58 (±1.06)	12 [12.00-13.00]		
Marital status**	Married	13.20 (±1.35)	13 [12.00-14.00]	12.82 (±1.17)	12 [12.00-13.00]	0.440; test=1.644	0.678; test=0.776
	Divorced	13.66 (±1.03)	13 [13.00-15.00]	13.00 (±1.10)	12 [12.00-13.50]		
	Widowed	13.00 (±0.81)	13 [12.00-14.00]	13.00 (±1.25)	12.5 [12.00-14.00]		

Clinical factors associated with medication adherence

Among the clinical characteristics assessed, the presence of diabetes mellitus was the only factor significantly associated with medication adherence at six months postoperatively (p=0.025). Interestingly, diabetic patients demonstrated better adherence compared to non-diabetic individuals, as reflected by a lower median ARMS score of 12.00 (mean: 12.55 ± 0.77) versus 13.00 (mean: 13.20 ± 1.42) in non-diabetics. No significant difference was observed at three months (p=0.437), indicating that this association emerged over time.

No other clinical variables were found to be significantly associated with medication adherence at either time point. These findings suggest that clinical status or surgical complexity did not appear to influence medication-taking behavior in the early or intermediate postoperative period. Detailed information on the distribution of ARMS scores across clinical subgroups and the corresponding statistical comparisons are presented in Table 5.

**Table 5:** Association of clinical factors with medication adherence at three and six months postoperatively Statistical tests: *Mann-Whitney U test; **Kruskal-Wallis H test. LVEF, left ventricular ejection fraction

Variable	Category	3 months, mean (SD)	3 months, median [IQR]	6 months, mean (SD)	6 months, median [IQR]	p-value and test (3 months)	p-value and test (6 months)
LVEF**	LVEF ≤40%	13.29 (±1.31)	13 [12.00-14.00]	13.53 (±1.74)	13 [12.00-14.00]	0.914; test=0.181	0.107; test=4.471
	LVEF 41-50%	13.11 (±1.16)	13 [12.00-14.00]	12.77 (±0.97)	12 [12.00-13.00]		
	LVEF >50%	13.30 (±1.47)	13 [12.00-14.00]	12.62 (±0.93)	12 [12.00-13.00]		
History of myocardial infarction*	Yes	13.23 (±1.20)	13 [12.00-14.00]	12.58 (±0.79)	12 [12.00-13.00]	0.782; test=652	0.449; test=607
	No	13.21 (±1.32)	13 [12.00-14.00]	12.90 (±1.21)	12 [12.00-13.00]		
Diabetes*	Yes	13.04 (±0.99)	13 [12.00-14.00]	12.55 (±0.77)	12 [12.00-13.00]	0.438; test=1,063	0.025; test=883
	No	13.43 (±1.58)	13 [12.00-14.00]	13.20 (±1.42)	13 [12.00-14.00]		
Type of surgery*	CABG	13.30 (±1.39)	13 [12.00-14.00]	12.86 (±1.10)	12 [12.00-14.00]	0.507; test=987	0.760; test=1,034
	CABG+valve	13.06 (±1.13)	13 [12.00-14.00]	12.82 (±1.27)	12 [12.00-13.00]		
Number of grafts**	1	13.28 (±1.18)	13 [12.00-14.25]	12.61 (±0.91)	12 [12.00-13.00]	0.793; test=1.034	0.580; test=1.962
	2	13.00 (±1.06)	13 [12.00-14.00]	13.00 (1.27±)	13 [12.00-13.50]		
	3	13.43 (±1.40)	12 [12.00-14.00]	12.71 (±1.05)	12 [12.00-13.00]		
	4	13.35 (±1.73)	12.5 [12.00-15.00]	13.07 (±1.38)	12 [12.00-14.00]		
Presence of arrhythmias*	Yes	12.96(±1.07)	13[12.00-14.00]	13.04 (±1.22)	13 [12.00-14.00]	0.314; test=804.5	0.245; test=792.5
	No	13.30(±1.37)	13[12.00-14.00]	12.77 (±1.34)	12 [12.00-13.00]		

## Discussion

Medication adherence after CABG is a critical factor for improving prognosis and reducing complications, as has been documented in the literature [5,8,12]. In the present study, a statistically significant improvement in adherence was observed at six months compared to three months postoperatively, indicating better medication adherence over time. The recorded improvement is considered clinically significant, as indicated by the fact that the proportion of patients with high adherence increased from 37.1% at three months to 50.5% at six months. These findings are comparable to international data, where rates of high adherence after CABG range between 40% and 60% at 6-24 months, while the proportion of patients with sub-optimal adherence, defined as moderate or low adherence according to the ARMS classification, remains a major challenge globally [5,13,14]. It is important to note that, in the context of this study, no cases of low adherence were recorded at any follow-up point.

Despite the improvement, nearly half of the patients continued to exhibit a moderate level of adherence at six months, highlighting the need for further strengthening of therapeutic consistency, particularly during the critical first six months after surgery. The initially moderate adherence observed in more than 60% of patients at three months may result not only from the complexity of new medication regimens and multiple prescribing physicians but also from patients’ limited oversight of their overall drug intake. Improved clinical practices, including enhanced patient education and monitoring, could help optimize adherence rates in Cyprus [14,15]. The gradual improvement at six months may be attributed to the patients’ natural adaptation, increased familiarity with the treatment regimen, and the development of personal management strategies, such as reminders and better medication organization. Additionally, repeated consultations with cardiologists likely reinforce the importance of adherence, helping patients understand why consistent medication use is critical [16,17].

The improvement in adherence was observed in the absence of any structured interventions, which may support the notion that undergoing CABG surgery represents a critical teachable moment for the initiation of positive health behaviors [16]. Nevertheless, existing literature emphasizes that the long-term maintenance of medication adherence typically requires multifaceted interventions. These include structured patient education, nurse-led guidance on proper and consistent medication use, and the integration of digital health technologies, such as electronic reminder systems and mobile health applications. Notably, multidimensional interventions that combine behavioral, educational, and technological components have been shown to be more effective than isolated strategies [18,19].

Existing literature presents mixed findings regarding the role of gender in medication adherence. Some studies suggest that women may exhibit greater awareness or sensitivity toward medical instructions [20-22], while others report no significant gender differences or even lower adherence among women, particularly in specific medication categories [23,24]. It is important to note, however, that the majority of these studies focus on general or mixed clinical populations rather than individuals undergoing CABG. In addition, variations in comorbidities, health beliefs, and health literacy between genders may further influence adherence and partly explain the observed results. This highlights a knowledge gap in the literature, as limited evidence is available on gender-related adherence patterns specifically within the post-CABG context. Moreover, the relatively narrow range of ARMS scores in our sample may have limited the ability to detect subtle differences across subgroups, suggesting that future studies with larger and more diverse cohorts are needed to fully elucidate these associations. The findings of the present study demonstrating better adherence among male patients therefore contribute meaningfully to this underexplored area and warrant further investigation in larger, longitudinal cohorts.

 A systematic review and meta-analysis of 28 studies involving patients with acute coronary syndromes found that women were less adherent to lipid-lowering therapies, whereas no significant gender differences were reported for antiplatelet agents or ACE inhibitors [24]. This variability in adherence patterns across medication classes underscores the complexity of medication-taking behavior and may be influenced by cultural norms, gender roles, perceived treatment benefits, and psychological factors [20,25]. While the present study did not assess adherence by drug class, the observed gender differences in overall adherence contribute to this ongoing discussion and suggest the need for further investigation into class-specific patterns of medication use, particularly in post-CABG populations.

The higher adherence observed among diabetic patients in this study appears to diverge from prior research, which often reports lower adherence rates in individuals with chronic diseases, including diabetes mellitus. For example, population-based studies have found that between 43% and 57% of diabetic patients exhibit low adherence to long-term medication regimens [26,27]. This discrepancy may be explained by contextual differences in study populations and timing: the present cohort consisted of patients recovering from a major cardiac surgery, where heightened motivation for behavioral change may be more prevalent. In this context, patients with pre-existing experience in managing complex medication schedules, such as those with diabetes, may have been better prepared to adapt to postoperative treatment demands, resulting in higher adherence. Moreover, it is possible that diabetic patients in this cohort were already more engaged in their health care due to routine follow-ups and long-term self-management, which may have fostered greater awareness and consistency in medication-taking behavior.

In contrast, no statistically significant associations were observed for variables such as age, educational level, type of surgery, or number of grafts in relation to medication adherence. These findings align with some prior studies, while differing from others in which factors such as age and education have been identified as significant predictors of adherence behaviors [5,13,14]. One possible explanation for this discrepancy is the relatively small sample size of the present study, which may have limited the statistical power to detect modest associations. Moreover, the sociodemographic and clinical homogeneity of the study population may have reduced intergroup variability, making it more difficult to identify statistically significant differences across subgroups.

In summary, the findings of this study are partially consistent with international data regarding postoperative medication adherence rates and influencing factors. Notably, the higher adherence observed among diabetic patients and the lack of significant associations with sociodemographic characteristics reflect population-specific dynamics that may relate to features of the local healthcare context or cultural expectations surrounding recovery after cardiac surgery. The overall high-to-moderate level of adherence observed despite the absence of a structured intervention and the significant improvement noted between three and six months postoperatively underscores the potential motivational role of major surgical procedures in promoting positive health behaviors. However, in the absence of preoperative adherence data, this interpretation should be approached with caution. Nonetheless, these findings highlight the importance of implementing long-term, multifaceted interventions to support sustained adherence and optimize clinical outcomes over time.

Structured discharge planning, defined as a coordinated process that ensures patients receive comprehensive education, clear instructions, and individualized support for post-hospital care, personalized adherence counseling, and the use of digital reminder tools for patients with moderate adherence, is not currently incorporated into standard post-CABG care in Cyprus. Given that nurses play a pivotal role in discharge coordination, patient education, behavioral counseling, and the implementation of digital health solutions, prioritizing nurse-led interventions holds significant promise for improving adherence and care continuity. Implementing such strategies within a research framework may provide valuable insight into their potential to enhance adherence and enable assessment of their relative effectiveness. Future research should also explore patient perspectives through qualitative methods to better understand the barriers and facilitators to medication adherence in individuals undergoing CABG. Furthermore, longitudinal intervention studies comparing adherence support strategies such as mobile health technologies, nurse-led follow-up programs, or behavioral counselling are warranted to inform the development of sustainable, patient-centered models specifically tailored to the needs of the CABG population.

This study has certain limitations. Medication adherence was assessed through self-report, which may introduce recall or social desirability bias. Additionally, the use of telephone interviews for data collection may have influenced participant responses due to potential reporting bias. The absence of baseline (preoperative) adherence data limits the ability to fully evaluate postoperative changes. The relatively small sample size, particularly among women, may have reduced the statistical power to detect associations. Furthermore, the six-month follow-up period does not allow for conclusions about long-term adherence patterns. In addition, the use of a convenience sampling method may limit the representativeness of the sample and introduce potential selection bias.

## Conclusions

In summary, this study demonstrates a statistically and clinically significant improvement in medication adherence during the first six months after CABG. Notably, better adherence was observed among diabetic patients and male participants, suggesting the influence of patient characteristics on postoperative behaviors. These findings highlight the importance of personalized strategies to support adherence. Future research should further investigate long-term adherence trajectories and evaluate context-appropriate interventions tailored to the needs of post-CABG populations.
